# Reproducibility of lung cancer radiomics features extracted from data-driven respiratory gating and free-breathing flow imaging in [^18^F]-FDG PET/CT

**DOI:** 10.1186/s41824-022-00153-2

**Published:** 2022-10-30

**Authors:** Daphné Faist, Mario Jreige, Valentin Oreiller, Marie Nicod Lalonde, Niklaus Schaefer, Adrien Depeursinge, John O. Prior

**Affiliations:** 1 Department of Nuclear Medicine and Molecular Imaging, Lausanne University Hospital, Rue du Bugnon 46, CH-1011 Lausanne, Switzerland; 2 Institute of Information Systems, University of Applied Sciences Western Switzerland, (HES-SO), Rue du Technopôle 3, CH-3960 Sierre, Switzerland; 3 Faculty of Biology and Medicine, University of Lausanne, Rue du Bugnon 21, CH-1011 Lausanne, Switzerland

**Keywords:** [^18^F]-FDG, Lung PET/CT, Data-driven respiratory gating, Radiomics features, Reproducibility, Respiratory gating, Data-driven gating, Radiomics features, PET/CT, Pulmonary nodule

## Abstract

**Background:**

Quality and reproducibility of radiomics studies are essential requirements for the standardisation of radiomics models. As recent data-driven respiratory gating (DDG) [^18^F]-FDG has shown superior diagnostic performance in lung cancer, we evaluated the impact of DDG on the reproducibility of radiomics features derived from [^18^F]-FDG PET/CT in comparison to free-breathing flow (FB) imaging.

**Methods:**

Twenty four lung nodules from 20 patients were delineated. Radiomics features were derived on FB flow PET/CT and on the corresponding DDG reconstruction using the QuantImage v2 platform. Lin’s concordance factor (*C*_*b*_) and the mean difference percentage (DIFF%) were calculated for each radiomics feature using the delineated nodules which were also classified by anatomical localisation and volume. Non-reproducible radiomics features were defined as having a bias correction factor *C*_*b*_  < 0*.*8 and/or a mean difference percentage DIFF% > 10.

**Results:**

In total 141 features were computed on each concordance analysis, 10 of which were non-reproducible on all pulmonary lesions. Those were first-order features from Laplacian of Gaussian (LoG)-filtered images (sigma = 1 mm): Energy, Kurtosis, Minimum, Range, Root Mean Squared, Skewness and Variance; Texture features from Gray Level Cooccurence Matrix (GLCM): Cluster Prominence and Difference Variance; First-order Standardised Uptake Value (SUV) feature: Kurtosis. Pulmonary lesions located in the superior lobes had only stable radiomics features, the ones from the lower parts had 25 non-reproducible radiomics features. Pulmonary lesions with a greater size (defined as long axis length > median) showed a higher reproducibility (9 non-reproducible features) than smaller ones (20 non-reproducible features).

**Conclusion:**

Calculated on all pulmonary lesions, 131 out of 141 radiomics features can be used interchangeably between DDG and FB PET/CT acquisitions. Radiomics features derived from pulmonary lesions located inferior to the superior lobes are subject to greater variability as well as pulmonary lesions of smaller size.

**Supplementary Information:**

The online version contains supplementary material available at 10.1186/s41824-022-00153-2.

## Background

Lung cancer diagnosis, staging and recurrence follow-up depend on 2-deoxy-2-[^18^F]fluoro-D-glucose PET/CT imaging as a key step (Lardinois et al. 2003; Keidar et al. 2004). The precision of mass contouring on PET/CT is limited by the acquisition time that covers several respiratory cycles. Indeed, volumes located close to the diaphragm are subject to cranio-caudal movements and show on free-breathing PET/CT an overestimation of delimited volumes in addition to a decrease of the maximum standardised uptake value (SUV_max_) (Nehmeh et al. 2002; Goerres et al. 2002). Furthermore, the different time spans for PET and CT acquisitions generate a misalignment in the superposition of both images as well as an attenuation imprecision (Visvikis et al. 2003). In order to use lung [^18^F]-FDG PET/CT in all its potential advanced implementations, respiratory artifact correction is fundamental (Farwell et al. 2014). Data-driven respiratory gating (DDG) is a recently introduced method that retrospectively derives respiratory motion signal from the detected movements of internal structures. The images are taken in numerous short sequences and are reconstructed (Schleyer et al. 2009). DDG PET/CT shows a superior performance to external device-based gating and could be widely implemented in clinical routine for lung lesion characterisation (Walker et al. 2020).

Besides the respiratory movements, a general limitation in medical imaging is the discrepancy between the amount of information available on images and the quantity perceived by a physician. Considering that the human eye can differentiate 900 shades of grey while CT images can have up to 65′536, only a small amount of information from PET/CT images is read (Kimpe and Tuytschaever 2007). Radiomics is a recent tool that enables the use of thorough information by deriving numerous predefined quantitative radiomics features. Those describe a large range from basic statistical image signal to complex texture characteristics (Lambin et al. 2012). The goal of radiomics is to develop algorithms that give advanced clinical and predictive information to perform personalised medicine (Meng et al. 2019). Lung cancer radiomics has already shown promising perspectives in classification of lung nodules, determination of histology, genomic characteristics and treatment outcome prediction (Ayachy et al. 2021). However, quality and reproducibility of radiomics studies remain essential considerations for the advance of radiomics models (Lambin et al. 2017). The aim of this study was to evaluate the impact of DDG on the reproducibility of radiomics features derived from [^18^F]-FDG PET/CT in comparison to free-breathing (FB) flow imaging.

## Materials and methods

All the patients participating in this retrospective study have signed a general consent form from the CHUV for research and for retrospective use of their images for clinical research. A protocol was submitted to CER-VD (protocol radiomics 2018–01513). Patients addressed for initial staging of lung cancer were included based on the following size criteria for pulmonary lesions: minimum long axis length of 10 mm and maximum long axis length of 64 mm. The [^18^F]-FDG PET/CT scans were taken between August 2020 until January 2021.

### [^18^F]-FDG PET/CT acquisition

Patients underwent [^18^F]-FDG PET/CT on a Siemens Biograph Vision 600 (Siemens Healthineers) with a 26.2 cm axial PET field-of-view. Image acquisition started one hour after a 2 MBq/kg intravenous inject ion of [^18^F]-FDG. All patients had fasted for at least 6 h and had blood glucose levels lower than 140 mg/dL before administration of [^18^F]-FDG. A low dose CT was first performed for anatomic correlation and attenuation correction (100 Ref mAs; 100 kV; CARE Dose4D; CARE kV; pitch, 1; time per rotation, 0.8 s; slice thickness, 2 mm), followed by a whole-body continuous bed motion PET scan (1.4 mm/s). Acquired data were reconstructed with the vendor software, TrueX + TOF (UltraHD) algorithm employing 4 iterations and 5 subsets and an image matrix of 440 × 440 voxels. PET system applied time-of-flight information and the point-spread-function correction in the iterative reconstruction process. In addition, all pertinent image corrections (normalization, dead time, activity decay, random coincidence and attenuation and scatter corrections) were applied. Two PET images were reconstructed from the same data using the same acquisition time for all images: an ungated image without motion correction and an image with DDG respiratory gating (OncoFreeze AI, Siemens Healthineers). The respiratory motion compensation was employed for the whole-body acquisition. The motion compensated images in DDG derive the waveform directly from the acquired continuous bed motion PET raw data (Buther et al. 2020; Schleyer et al. 2018).

### Clinical evaluation

**Fig. 1:**
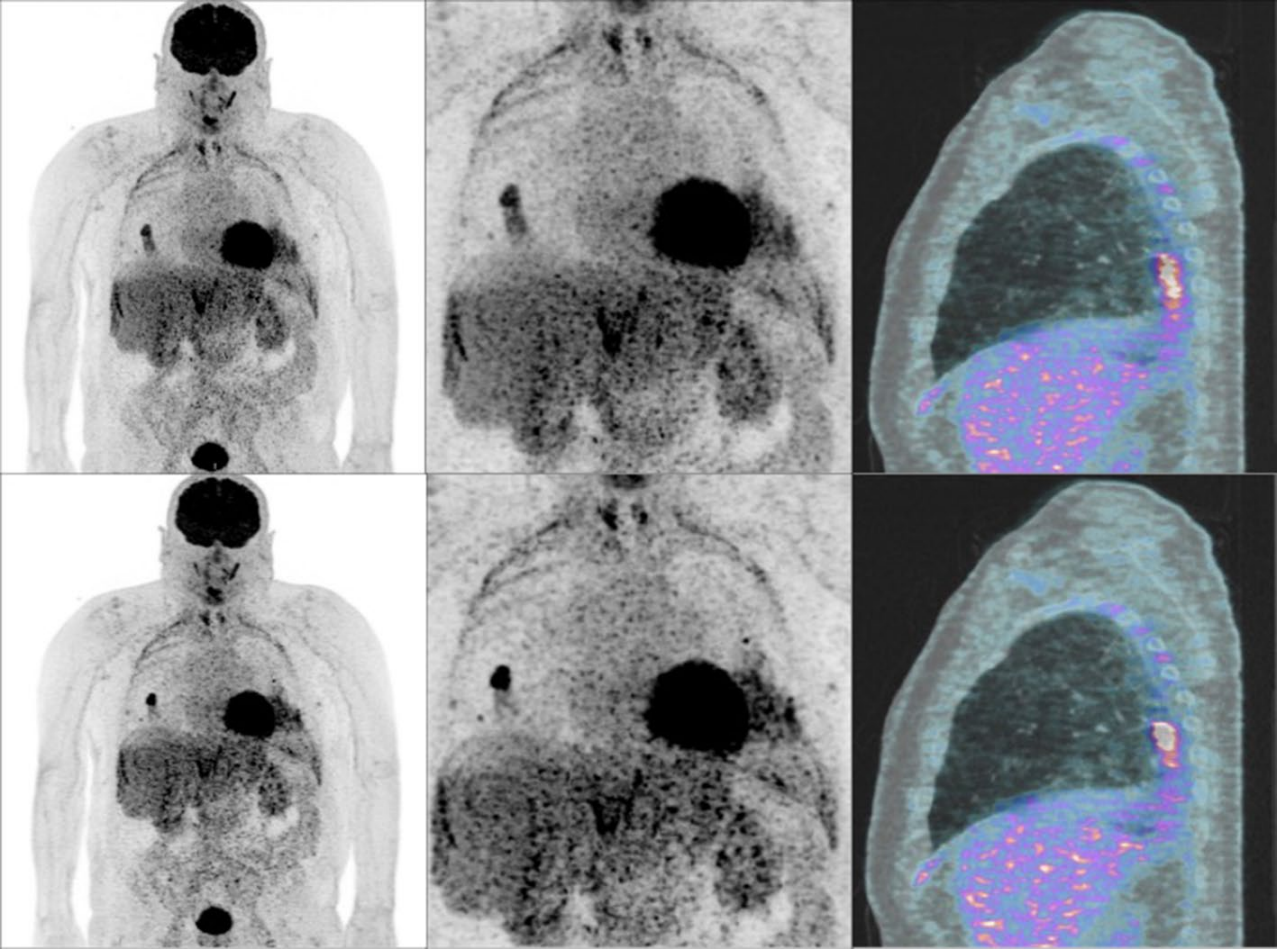
Free-breathing flow (first row) vs data-driven respiratory gating (second row) ^18^F-FDG PET/CT

On the [^18^F]-FDG PET/CT, one or two pulmonary lesions per patient were delineated with the same volume of interest (VOI) defined by a 42% threshold of the SUV_max_ for the two reconstructions. In total 24 lung nodules were delineated from 20 patients. The difference in size and shape of the lung nodule between FB and DDG PET/CT reconstructions is illustrated in Fig. 1. The VOI were extracted from each [^18^F]-FDG PET/CT and uploaded to the QuantImagev2 platform (Schaer et al. 2022). Radiomics features were subsequently derived for the two reconstructions of each VOI (Cid et al 2017; Aerts et al. 2014; Ravanelli et al. 2013).

### Statistical analysis

**Fig. 2:**
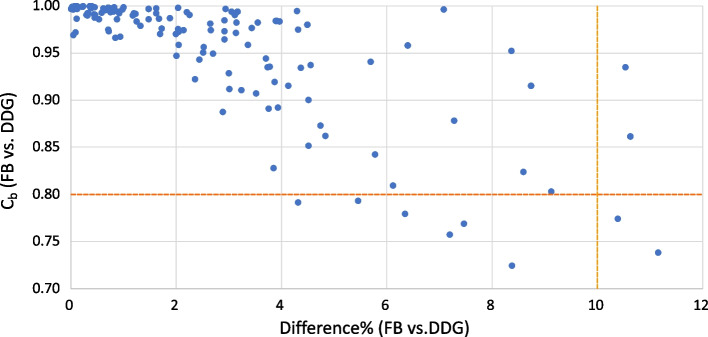
Distribution of *C*_*b*_ and DIFF% comparing radiomics features from DDG and FB images

To compare features from the two acquisitions, we calculated Lin’s concordance factor (*ρ*_*c*_) which is equal to the Pearson correlation coefficient multiplied by the bias correction factor (*C*_*b*_) (Lawrence and Lin 1989). In addition, we calculated the mean difference percentage (DIFF%) between the two imaging methods. The resulting *C*_*b*_ and DIFF% values distribution calculated on all pulmonary lesions is represented in Fig. 2. Non-reproducible features were defined as having a *C*_*b*_ < 0*.*8 and/or a DIFF% > 10. To evaluate a difference depending on the lesions localisation two separate groups were defined: lesions in the superior lobes (13 lesions) versus the others (11 lesions), on which *C*_*b*_ and DIFF% were calculated separately. The pulmonary lesions were also dichotomised in two groups based on their size: long axis length shorter (12 lesions) or longer (12 lesions) than the median, on which *C*_*b*_ and DIFF% were calculated separately.

## Results

We retrospectively analysed 24 pulmonary lesions from 20 patients (10 female, 10 male) with mean age of 70.8 ± 6.3 years. The lesions were located in the superior lobes (right 5/24, left 8/24), lingula (1/24) and inferior lobes (right 6/24, left 4/24), had a long axis median of 26.5 mm, a Metabolic Tumour Volume of 68.5 ± 90.6 (g) and a SUV_max_ (g/ml) of 19.8 ± 7.7 on DDG and 17.0 ± 7.5 on FB reconstructions. Eventually, the resulting histology diagnosis for the patients were Adenocarcinoma (12/20), Squamous Cell Lung Cancer (4/20), Non-Small Cell Lung Cancer (2/20), Small Cell Lung Cancer (2/20).

In total, 141 radiomics features were computed and their values are detailed in Additional file 1: Table S1. Out of these 141 radiomics features, 131 (92.9%) showed stable reproducibility indicating that they are not affected by respiratory movements. The reproducibility results between FB and DDG PET/CT in terms of percentage difference and bias coefficient *C*_*b*_ are tabulated in Additional file 2: Table S2 for each of the 141 radiomics features. Important differences between FB and DDG PET/CT were observed on 10 features (7.1%) that were defined as non-reproducible. Table 1 lists the non-reproducible features with their respective *C*_*b*_ and DIFF% values and implicates first-order SUV features (1/18, 5.6%), texture features from Gray Level Cooccurence Matrix (GLCM) (2/22, 9.1%) and mainly shape features from first-order of Laplacian of Gaussian (LoG)-filtered images (sigma = 1 mm) (7/85, 8.2%). By doing the same reproducibility analysis in two separate groups on the pulmonary lesions depending on their upper or lower localisation, 25 features (17.7%) were considered as non-reproducible for lower localisation opposed to none from the superior lobes. For the 141 radiomics features DIFF% and *C*_*b*_ values are listed for the two lesion groups in Additional file 3: Table S3. When calculating radiomics features reproducibility on two lesions groups sorted by their size (long axis length < or > than the median), 20 /141 (14.2%) features extracted from the smaller lesions were defined as non-reproducible compared to 9/141 (6.4%) features for the bigger lesions. The reproducibility values DIFF% and *C*_*b*_ on the small and big lesion group are tabulated in Additional file 4: Table S4.

**Table 1:** List of non-reproducible features from all lesions

SUV features	DIFF%	*C*_b_
SUV_Kurtosis	6.3422041	**0.779951236**

GLCM features		
glcm_ClusterProminence	**10.532818**	0.935542057
glcm_DifferenceVariance	**10.38578**	**0.774378898**

Log-sigma features		
log-sigma-1–0-mm_Energy	**10.618035**	0.861606013
log-sigma-1–0-mm_Kurtosis	7.1899033	**0.757987361**
log-sigma-1–0-mm_Minimum	8.3714485	**0.724717193**
log-sigma-1–0-mm_Range	7.466568	**0.769357684**
log-sigma-1–0-mm_RootMeanSquared	5.4554815	**0.793799489**
log-sigma-1–0-mm_Skewness	4.3091531	**0.791736824**
log-sigma-1–0-mm_Variance	**11.146678**	**0.738479189**

Bold typeface indicates which value from DIFF% and/or *C*_b_ are non reproducible (DIFF>10%, *C*_b_<0.80)

## Discussion

We retrospectively evaluated the reproducibility of radiomics features derived from FB and DDG [^18^F]-FDG PET/CT. Most radiomics features (131/141) showed strong reproducibility except for 10 features that were considered as non-reproducible. Reproducibility was mainly compromised on LoG filters with sigma = 1 mm. Interestingly, LoG features with sigma filter size greater than 1 mm were reproducible indicating that respiratory blurring is not detected with filters of ≥ 2 mm. There was only one outlier out of 19 SUV features. Low variability in SUV features is important because this category is frequently used, for instance in lymphoma treatment assessment as part of the Deauville score (Meignan et al. 2009). In the clinical study of Dias et al. (Dias et al. 2022) a quantitative difference in SUV features values between DDG and belt-gated PET/CT was found. Despite the quantitative aspect there were not any changes on the clinical outcomes. Their study showed potential impact of precise respiratory gating for quantitative outcomes and how DDG could improve clinical decisions based on quantitative algorithms.

Furthermore, in our study we compared the reproducibility according to the anatomical localisation. Lesions located in the superior lobes had exclusively features with high reproducibility, as opposed to features from lower regions which had high variability. This discrepancy can be explained by the respiratory movements, which result in a greater displacement of the lower parts of the lungs closer to the diaphragm (Grootjans et al. 2014). In addition to the localisation, we evaluated reproducibility of radiomics features depending on pulmonary lesions size. Smaller lesions had more than twice as many non-reproducible features than bigger lesions. This result can be explained by the fact that respiratory blurring impacts a greater fraction of a smaller lesion and its radiomics features are exposed to greater variability.

To our knowledge, other studies have evaluated radiomics features behaviour under different image segmentation, discretisation and reconstruction methods but have not focused on their reproducibility. Xu et al. (2021) measured respiratory motion influence on radiomics features stability with phantom simulation of different respiratory patterns. Based on the different radiomics values distribution, the stability of the features was evaluated calculating the coefficient of variation and the relative difference. In contrast to our reproducibility study, only a small fraction of radiomics features was considered as stable because radiomics features had varying values between the different respiratory patterns. Yamashita et al. (2021) only looked at texture features derived from step-and-shoot and continuous bed motion [^18^F]-FDG PET/CT. To see the influence between the two acquisitions, the mean difference percentage and correlation coefficient between the two derivations of radiomics features were calculated. Although the study is similar, Lin’s concordance and the corresponding reproducibility was not evaluated. However, in their results they considered the entropy of the GLCM matrices as the most adapted feature for clinical use. The high reproducibility of this feature is consistent with our results which have very stable DIFF% and *C*_*b*_ values for all GLCM entropy features.

There are two important aspects regarding the clinical implications of this study. First, we concluded that most radiomics features have a strong reproducibility and can be used interchangeably on both DDG and FB PET/CT. We encourage further use of those features regardless of the respiratory motion reconstruction. As to the features considered as non-reproducible, they cannot be used interchangeably with FB or DDG PET/CT and should only derive from DDG images where the respiratory motion is controlled. Special attention should be paid to algorithms based on non-reproducible features. Its features should not be derived from FB acquisitions as the algorithms’ outcome will most probably not be reliable.

Our study design has some limitations. First, this was a single-centred and retrospective study depending on the same PET/CT scan for all acquisitions. Common variability between acquisitions was not included and its presence may have changed the reliability of other features. On the other hand, using the same PET/CT scan excluded the detection of machine-generated bias. Because CT images are not gated, its radiomics features were not included and this limited our considerations to PET features only. Finally, the non-reproducible features were defined by choosing thresholds from the graph (Fig. 2) with reproducibility values *C*_*b*_ and DIFF% but depending on the accepted variability, they could have been defined differently.

Despite those limitations, calculating Lin’s concordance with bias correction factor *C*_*b*_ enabled the detection of a lack of concordance within the correlation and permitted a correct evaluation of the quality and reproducibility of radiomics features. Those aspects are very important as machine-learning algorithms are based on radiomics features that must strictly avoid biases. Using QuantImage v2 is also a strength of this study as it is an open-source platform which enables further standardisation of radiomics features, a key step for wide standardisation and implementation of radiomics (Welch et al. 2019).

## Conclusion

In this retrospective study, most radiomics features (131/141) can be used interchangeably with DDG or FB as they showed high reproducibility. Our results suggest that the 10 radiomics features with highest variability are affected by FB and should only be used with DDG images. Radiomics features derived from pulmonary lesions located inferior to the superior lobes are subject to greater variability as well as pulmonary lesions of smaller size.

## Supplementary Information

**Additional file 1.** Individual radiomics features for the free-breathing and data-driven gating PET/CT data sets. Individual radiomics features values for the free-breathing (flow) and data-driven gating (DDG) PET/CT reconstruction of each pulmonary lesion. The radiomics features table is the output from the QuantImage platform (see Cid et al. (2017)).

**Additional file 2.** Differences and concordance among free-breathing and data-driven gating PET/CT for all radiomics features. The relative difference in percentage (DIFF%) and the concordance bias coefficient (Cb) are presented for each of the 141 radiomics features as derived from the QuantImage Excel output (see Cid et al. (2017)).

**Additional file 3.** Differences and concordance among free-breathing and data-driven gating PET/CT for all radiomics features calculated separately based on pulmonary lesions localisation. The relative difference in percentage (DIFF%) and the concordance bias coefficient (Cb) are presented for each of the 141 radiomics features derived separately on each group “superior” and “inferior” depending on their anatomical localisation: lesions in the superior lobes versus the inferior regions. Non-reproducible features are marked with a star.

**Additional file 4.** Differences and concordance among free-breathing and data-driven gating PET/CT for all radiomics features calculated separately based on pulmonary lesions size. The relative difference in percentage (DIFF%) and the concordance bias coefficient (Cb) are presented for each of the 141 radiomics features derived separately on two lesions groups “big” and “small” depending on their long-axis length < or > median. Non-reproducible features are marked with a star.

## Data Availability

The datasets supporting the conclusions of this article are included within the article and its additional files. The supplementary files include additional files 1, 2, 3, 4. Table S1: Radiomics features derived from each DDG and FB reconstruction. Table S2: Differences and concordance among free-breathing and data-driven gating PET/CT for all radiomics features. Table S3: Differences and concordance among free-breathing and data-driven gating PET/CT for all radiomics features calculated separately based on pulmonary lesions localisation. Table S4: Differences and concordance among free-breathing and data-driven gating PET/CT for all radiomics features calculated separately based on pulmonary lesions size.

## Abbreviations

*C*_*b*_: Bias correction factor from Lin’s concordance
DDG: Data-driven gating
DIFF%: Mean difference percentage
FB: Free-breathing
SUV: Standardised uptake value
